# A social contagious model of the obesity epidemic

**DOI:** 10.1038/srep37961

**Published:** 2016-11-28

**Authors:** He Huang, Zhijun Yan, Yahong Chen, Fangyan Liu

**Affiliations:** 1 School of Management and Economics, Beijing Institute of Technology, Beijing 100081, China

## Abstract

Obesity has been recognized as a global epidemic by WHO, followed by many empirical evidences to prove its infectiousness. However, the inter-person spreading dynamics of obesity are seldom studied. A distinguishing feature of the obesity epidemic is that it is driven by a social contagion process which cannot be perfectly described by the infectious disease models. In this paper, we propose a novel belief decision model based on the famous Dempster-Shafer theory of evidence to model obesity epidemic as the competing spread of two obesity-related behaviors: physical inactivity and physical activity. The transition of health states is described by an SIS model. Results reveal the existence of obesity epidemic threshold, above which obesity is quickly eradicated. When increasing the fading level of information spread, enlarging the clustering of initial obese seeds, or introducing small-world characteristics into the network topology, the threshold is easily met. Social discrimination against the obese people plays completely different roles in two cases: on one hand, when obesity cannot be eradicated, social discrimination can reduce the number of obese people; on the other hand, when obesity is eradicable, social discrimination may instead cause it breaking out.

Obesity, also known as “globesity”, has become a worldwide health problem^1^. Its rapid growth and sometimes mortality risks^2^ have aroused more and more public awareness, but controlling it is not an easy task. Understanding its inter-person dynamics is considerably important.

Social environment, especially social relationships, are closely correlated with people’s health. A lack of social connections may increase the odds of death^3^. In a social network, many phenomena have been found to spread interpersonally, such as biological games^4^, indirect reciprocity^5^, misinformation or rumors^6^,^7^, and infectious diseases^8^,^9^. Obesity is one of them^10^ and is often seen as a contagious epidemic^1^. It is therefore reasonable to model the spread of obesity using a mathematical epidemic model. Hill *et al*. tried to adopt an SIS model to describe obesity as a special “infectious disease”^11^. However, the spread of obesity is much different from the traditional biological viral contagions. The most distinguishing feature is that it is not driven by the interpersonal contact. Diverse mental factors are predisposing to obesity, like depression, self-esteem, sense of security, etc^12^,^13^, but they are often overlooked^14^. Since emotions can be transferred directly from one individual to another by mimicry or “emotional contagion”^15^,^16^, these mental factors can be modeled into a social contagion process^17^. In other words, the inter-person spreading dynamics of obesity are driven by a social contagion process.

To the extent that obesity is a product of voluntary behaviors^10^, the obesity epidemic often results from the spread of undesirable health-related behaviors, such as binge eating, while preventing the contagion of healthy behaviors at the same time. As summarized by WHO^1^, the behaviors which can increase weight, such as television watching, working on a computer, etc, are classified as physical inactivity or sedentary behaviors, and those behaviors which have the opposite effects, like exercising and sporting, are named physical activity. Hill and Peters claimed that “the epidemic of obesity is caused largely by an environment that promotes excessive food intake and discourages physical activity”^18^. A lifestyle with insufficient physical activity and excessive inactivity often causes an individual gaining weight^19^. By engaging in regular physical activities, people’s BMI can be reduced^20^,^21^ and obesity may be prevented^14^. Physical activity and inactivity can often coexist in people’s lifestyles but rival to each other. When physical inactivity holds a dominant position, a healthy person may gain weight gradually. Conversely, when physical activity occupies the leading position, an obese person may lose weight and finally regain a healthy weight.

Therefore, the social contagion process of obesity can be divided into two steps: First, whether does an individual adopt physical inactivity or physical activity? Second, whether does the individual become obese by physical inactivity or regain a healthy weight by physical activity? A typical SIS model can only catch the second step while ignoring the first one. The modeling of competing behaviors requires the decision maker to make judgments on a number of decision alternatives. Moreover, people’s subjective preferences for behaviors are not quickly determined but changed bit by bit. These make it difficult to model the first step of the social contagion process. A wonderful solution is the belief decision model which is developed based on the framework of Dempster-Shafer theory of evidence (DST). The most important feature of this model is to update people’s beliefs for multiple options after they receive various information bit by bit from different sources in the presence of uncertainty^22^. The term “belief” is used to denote people’s preferences and propensities to behaviors, thus the obesity-related mental factors can be quantified by this index. This notion is in line with a stream of decision-making researches which suggest that people are influenced by their prior beliefs and expectations when evaluating new information^23^. Using the belief decision model, Xia and Liu^22^ successfully characterized the spread of competing awareness during an infectious disease course. Therefore, we combine the belief decision model with the SIS model to depict the social contagion process of obesity.

Our results echoes the findings of Hill *et al*.^11^ that the obesity epidemic reaches equilibrium as time passes by. Moreover, we discover that, the social contagion of obesity can be contained and even eradicated through the competing spread of physical activity belief and physical inactivity belief. The thresholds for eradicating obesity are dependent on many factors, such as the network topology and the clustering of the initially obese seeds. In addition, we study the influence of social discrimination on the obesity epidemic, and find it generates distinct outcomes under different conditions.

## Results

Agent-based simulation is an appropriate tool to explore theoretical epidemic dynamics in a population within complex network structures. The topological distribution of individuals on a population’s social network strongly affects the probability of an epidemic^17^. We adopt the homogeneous Watts-Strogatz model to include a wide range of network topologies with varying rewiring probability, *p*_*r*_^24^. This model allows us to capture highly structured ring-lattices (small *p*_*r*_), highly unstructured random graphs (large *p*_*r*_) and a variety of small-world network topologies (medium *p*_*r*_). With the increase of *p*_*r*_, the networks become less structured but of more randomness, which is very influential on the spreading process^25^. The spread of obesity is modeled on the networks of *N* = 1000 individuals with average degree 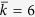. As shown in the “Methods” section, people have three decision alternatives: to be physical inactive (*x*), to be physical active (*y*) and undecided (or hesitating) for a behavior change (*θ*). Each is attached with a belief mass to denote people’s subjective preferences towards the alternative. For initialization, we set a proportion (10%) of the nodes to be obese seeds and take physical inactivity with the belief mass {*m(x*), *m(y*), *m(θ*)} = {1, 0, 0}. The rest nodes are set to be healthy. We then divide the healthy people into two groups: 1/9 (10% of the whole population) to be physical active with the belief mass {0, 1, 0}, 8/9 to be completely hesitating with the belief mass {0, 0, 1}.

We use *ρ*_*o*_ to denote obese density, *ρ*_*h*_ to denote healthy density, *ρ*_*p*_ to denote physical active density, and *ρ*_*ip*_ to denote physical inactive density. As shown in ref. 11, the efficiency of a healthy person spontaneously becoming obese is 0.02, thus we derive the efficiency of physical inactivity *ω*_1_ as 0.02. Similarly, the physical active efficiency *ω*_2_ is derived as 0.04. For the spread of belief, we propose a fading coefficient *η* to describe how fast information decays when spreading in the network. If the fading coefficient *η* grows, people are faced with more obstacles to learn from their peers and are less affected. The fading coefficient is derived from the study of Xia and Liu^22^ as *η* = 0.2. Moreover, we also consider a reasonable range of it.

**Figure 1:**
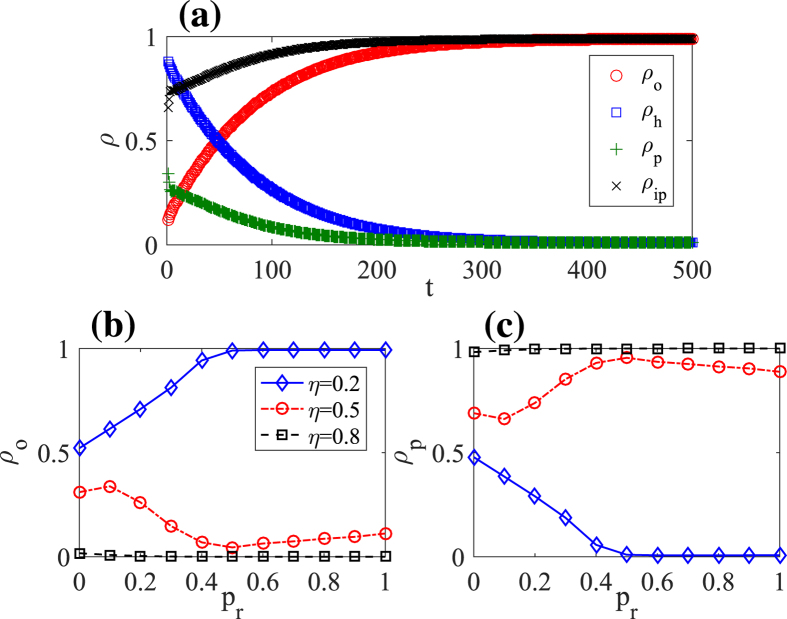
(**a**) A typical spreading case of obesity and the related behaviors. The effects of the fading coefficient of information spread *η* on (**b**) the obese density *ρ*_*o*_ and (**c**) the physical active density *ρ*_*p*_ with the network rewiring probability *p*_*r*_ ∈ [0, 1]. Each data in this figure and figures below are averaged over 500 independent runs. The parameters here are: efficiency of physical inactivity (or ‘infection rate’) *ω*_1_ = 0.02, efficiency of physical activity (or ‘recovery rate’) *ω*_2_ = 0.04, fading coefficient of information spread *η* = 0.2, network rewiring probability *p*_*r*_ = 0.5.

Figure 1(a) shows that the system finally reaches a stationary state as time passes by. In this state, the system is nearly divided into two parts: one is obese and physical inactive, the other is healthy and physical active, which infers that all the obese people may finally take physical inactivity while the healthy people may take the physical activity to prevent obesity. Thus to eradicate obesity, well-formulated strategies are needed to spread the belief of physical activity. We then change the fading coefficient *η* to see how the spreading speed of belief affects the obesity epidemic. Increasing the fading coefficient, as seen from Fig. 1(b) and (c), can reduce the spread of physical inactive belief, correspondingly decreasing the obese density and sometimes eradicating it. It should be noted that, such eradication is dependent on a highly randomly distributed network topology which is efficient for information spreading. However, when the fading coefficient *η* is low, increasing network randomness instead increases the obesity epidemic. The difference infers that there may exist a threshold for the obesity epidemic.

### Threshold for obesity epidemic

**Figure 2:**
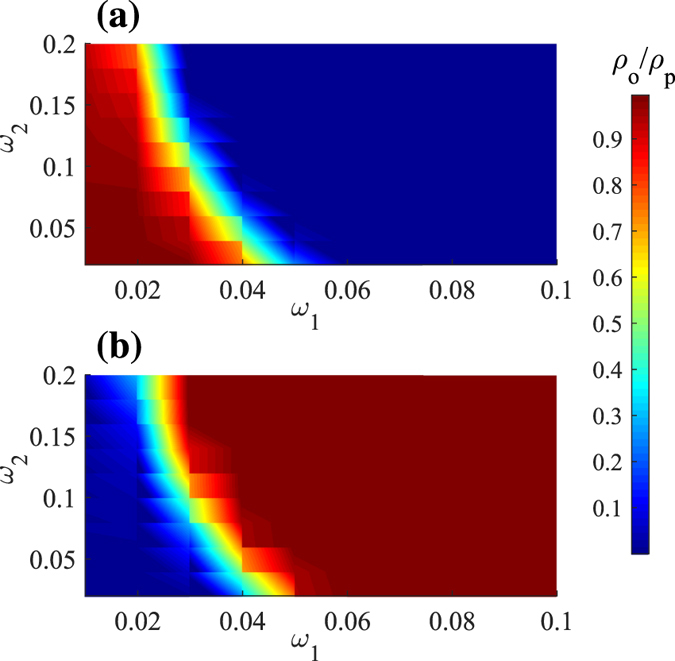
The evolution of (**a**) the obese density *ρ*_*o*_ and (**b**) the physical active density *ρ*_*p*_ in the parameter space *ω*_1_ − *ω*_2_. The value of fading coefficient of information spread *η* is 0.2 and the network rewiring probability *p*_*r*_ is 0.5. We delete the extreme values: *ω*_1_ = 0 and *ω*_2_ = 0.

Epidemic threshold is an important parameter in the modeling of infectious diseases. If the infectiousness of a disease is under the threshold, the epidemic is easily controlled and eradicated. During the obesity epidemic, people are continuously developing new efficient ways to reduce energy intake and lose weight. But the density of obese people still keeps increasing^26^. It is meaningful to explore whether the efficiency of physical activity or physical inactivity has the threshold. As seen from Fig. 2, the obesity density *ρ*_*o*_ and physical active density *ρ*_*p*_ are nearly complementary, verifying our above conclusion that all the healthy people will finally take physical activity. Moreover, as shown in Fig. 2(a), obesity finally dies out in the network with high values of both active and inactive efficiencies. Larger efficiencies correspond to faster eradication. Their thresholds are interdependent and interact together. If one of them has a very low efficiency (i.e. *ω*_1_ = 0.02), in order to eradicate the epidemic, the efficiency needed for another behavior may be very high (i.e. *ω*_2_ > 0.2).

Thus a more practical obesity-eradication strategy is to improve the efficiencies of both physical activity and inactivity, where increasing physical inactive efficiency may often be overlooked. It is somewhat surprising to get such a result because increasing physical inactive efficiency obviously makes people gain weight faster. However, when the physical inactive people gain weight at a slower speed, they will send physical inactive hints to their peers in a longer period. Conversely, a faster speed to become obese may instead prevent the spread of physical inactive belief, as seen from Fig. 2(b), thus reducing and even eliminating the obesity epidemic. To summarize, increasing the efficiency of physical active behaviors can make people return to a healthy weight more quickly and in turn promote the spread of physical active belief, while increasing physical inactive efficiency can prevent the spread of physical inactive belief. Both of them are essential and supplement each other.

### Initial obese clustering

**Figure 3:**
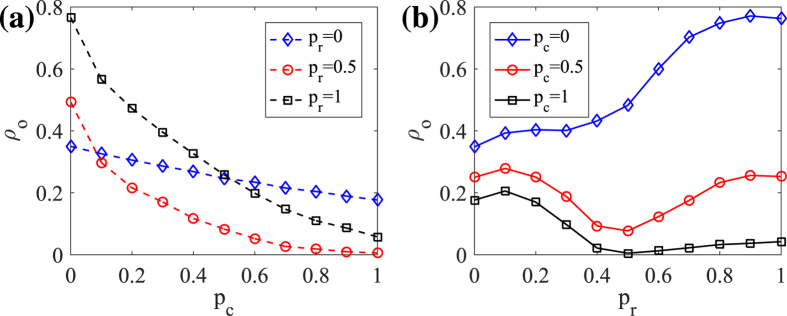
(**a**) The influence of the clustering of initially obese seeds *p*_*c*_ on the obesity epidemic *ρ*_*o*_ with three kinds of network topologies. (**b**) The influence of the network topology *p*_*r*_ on the obesity epidemic *ρ*_*o*_ with three values of the initial obese clustering. Other parameters here are: the physical inactive efficiency *ω*_1_ = 0.03, the physical active efficiency *ω*_2_ = 0.1, the fading coefficient of information spreading *η* = 0.2.

Opinions often occur in clusters, which can induce herd behavior^27^,^28^. The spread of physical inactive belief may make the initial obese seeds cluster together. Such opinion clusters can be highly influential on an epidemic spreading process^29^,^30^. Inspired by the sentiment contagion process proposed by Campbell and Salathe^17^, we propose a parameter *p*_*c*_ ∈ [0, 1] to denote the proportion of the initial obese people gathering in a cluster while the rest are randomly distributed in the network. If *p*_*c*_ = 0, all the initial obese people are randomly distributed. While *p*_*c*_ = 1, all the initial obese people gather in a single cluster. We also consider the network topology which affects the topology of the clustered initial obese people. To explore whether the obesity threshold is variable, we adopt the critical parameters of physical active and inactive efficiencies, namely *ω*_1_ = 0.03, *ω*_2_ = 0.1 from Fig. 2, for further analysis. In Fig. 3 we research the joint effects of the initial obese clustering and network topology on the obesity prevalence. A larger clustering proportion often leads to a lower obesity prevalence and even eradication. Because when the obese people cluster together, most of the physical inactive belief may be confined in the cluster and its influence on the healthy people is relatively low. Thus, a larger cluster size often indicates an easier and sometimes a faster eradication.

The network topology significantly affects the influence of clustering proportion *p*_*c*_ on the obesity prevalence, as shown in Fig. 3(a). In regular networks (*p*_*r*_ = 0), people are connected into clusters by strong ties^24^. It is hard for people in certain belief clusters (i.e. clusters connected by people with hesitant belief) to bring up a different belief (i.e. physical inactive belief). Thus regular networks are not only very resistant to spread obesity, but also reluctant to eliminate it. In random networks (*p*_*r*_ = 1), people are randomly connected by weak ties (or rewired edge)^31^,^32^, thus bringing up a different belief becomes relatively easy. For instance, obese people in random networks can contact with more unclustered people and induce them to take physical inactive behaviors, consequently resulting in the largest obesity prevalence when *p*_*c*_ = 0. Similarly, the obesity prevalence is rapidly decreased with *p*_*c*_ growing. In small-world networks (i.e. *p*_*r*_ = 0.5), there exist both clusters and weak ties^31^,^33^. The obesity density *ρ*_*o*_ when *p*_*c*_ = 0 is between that of regular and random networks. But the value of *ρ*_*o*_ keeps lower than that of random networks, inferring that the obesity prevalence is easier to contain in the small-world networks.

As shown in Fig. 3(b), the initial cluster proportion impacts the influence of network topology on the obesity prevalence, as well. When all the initial obese people are randomly distributed in the network (*p*_*c*_ = 0), the obesity prevalence grows with the rewiring probability *p*_*r*_. While those people are not completely randomly distributed (*p*_*c*_ = 0.5 or 1), the *ρ*_*o*_ − *p*_*r*_ curve nearly evolves in U-shape, verifying that obesity epidemic is easier to contain in small-world networks. On one hand, increasing rewiring links reduces network clusters and thus decreasing the resistant effect against the obesity invasion; on the other hand, larger rewiring probability increases the density of weak ties, which is beneficial to spread physical active belief into the obesity cluster. The small-world networks have both the advantages and are more efficient to spread information^34^.

### Social discrimination

Obesity is vulnerable to social bias and discrimination^35^,^36^,^37^ because it is often “highly stigmatized in terms both of the perceived undesirable bodily appearance and of the character defects that it is supposed to indicate”^1^. This may increase the social distance between the obese people and the rest healthy population. Inspired by the definition of “effective distance”^8^, we introduce a parameter *d* ∈ (0, ∞) to denote the social distance between obese and healthy people, where *d* = 1 indicates no discrimination; *d* > 1 indicates there is discrimination from the healthy people against their obese peers (positive discrimination); while *d* ∈ (0, 1) means that people are more willing to contact with the obese peers to, for instance, express their concern and care (negative discrimination).

**Figure 4:**
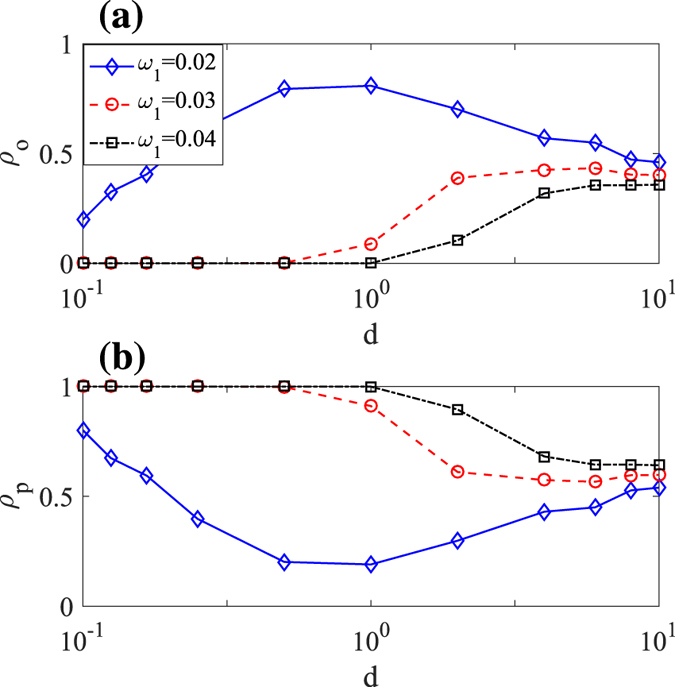
The influence of social discrimination on (**a**) the obese density *ρ*_*o*_ and (**b**) the physical active density *ρ*_*p*_. Other parameters are: *ω*_2_ = 0.1, *η* = 0.2, *p*_*r*_ = 0.5, *p*_*c*_ = 0.5.

Stronger social discrimination reduces the belief learning efficiency between healthy and obese people and prevents the spread of both physical active and inactive beliefs, while weaker social discrimination can accelerate their spread. Thus when obesity is ineradicable and the dominant belief is to take physical inactivity, it is reasonable to expect that larger social distance *d*, reduces more physical inactive belief than the physical active one, thus increasing the number of physical active people and correspondingly reducing the obese density. While obesity is eradicable and the physical active belief is at the dominant position, increasing social distance d, may decrease the number of physical active people and make the obese people remain obese^36^, correspondingly resulting in a severe obesity prevalence. In Fig. 4, we consider three conditions of the physical inactive efficiency: *ω*_1_ = 0.02, *ω*_1_ = 0.03, and *ω*_1_ = 0.04, where *ω*_1_ = 0.02 indicates that obesity is ineradicable at *d* = 1 and *ω*_1_ = 0.04 represents that obesity is eradicable at *d* = 1. The results when *d* > 1 verify our above analysis that stronger social discrimination reduces the obese density when obesity is ineradicable, but results in a severe obesity prevalence while obesity is eradicable.

However, when 0 < *d* < 1 and *ω*_1_ = 0.02, things are different. Although obesity is ineradicable in this case, it is interesting to find that decreasing social distance *d*, still reduces the obese density. Since weaker social discrimination accelerates the spread of beliefs, it becomes easier for the physical inactive people to bring up a physical active belief and be more susceptible to physical activity. Thus, decreasing social distance when 0 < *d* < 1 can instead enlarge the physical active density and reduce the number of obese people. This result infers that it may be more beneficial to eliminate social discrimination during the obesity epidemic.

## Discussion

Understanding the inter-person dynamics of obesity spreading is vital for the policy-makers to developing effective controlling strategies. However, obesity epidemic is different from the traditional biological contagion which relies on the spread of virus by direct social contact. Its spread depends on the diffusion of unhealthy behaviors: physical inactivity. To the extent that people’s behavior choices are influenced by their prior knowledge and expectations when receiving new information, we adopt the notion of belief to denote their subjective probabilities for a behavior. An extended belief decision model is proposed to describe the spread of physical activity and physical inactivity. This model is combined with a typical SIS model to describe the social contagion process of obesity in the WS networks. We hope our work can provide some heuristic influence on controlling obesity and modeling other social contagion phenomena.

The belief decision model is adopted to model the competence between two obesity-related behaviors, namely physical activity and physical inactivity, in the population. A person can select one of them based on the belief mass in every season and the behaviors may result in his health state transition. Such state change will produce new information and the person will transfer the information to his peers to update their beliefs. If a physical inactive person gains weight at a higher speed, he will have less time to transfer the physical inactive belief. If a physical active obese person regains a health weight more quickly, he will develop strong physical active belief and produce longer influence. Thus both high efficiencies of physical inactivity and activity can reduce and even eradicate the obesity epidemic. Moreover, the threshold efficiencies of both the behaviors to eradicate the obesity epidemic are discovered. Although the two behaviors are competing to each other, the threshold values are interdependent. The most important issue here is that even when the physical active efficiency is very high, a low physical inactive efficiency can still make the society tolerate the erosion of obesity.

Many factors can influence the eradication of obesity. In this work, we research the network topology, the decay of information, and the clustering of initial obese seeds. Results show that greater information decay or larger initial obese clustering often leads to lower obese prevalence and sometimes eradication. For the network topology, the small-world network is the easiest to eradicate obesity. Their effects are usually not independent but combine together.

Social discrimination against the obese people is a common phenomenon. Its existence often prevents the spread of information between healthy and obese people. As we understand, social discrimination will lengthen the path to transfer a piece of information and thus increasing the decay of information. We propose a social distance parameter to measure the discrimination level. Positive social distance reduces the learning efficiency of information but has two-sided effects on the obesity prevalence. When obesity is originally ineradicable, longer social distance helps reduce the obese density. While obesity is originally eradicable, increasing social distance instead enlarges the obese density and makes obesity survive. We also consider the situations where healthy people may express their concern and care to their obese peers, namely negative social distance. It is interesting and implicational to find that a more negative social distance decreases the number of obese people when obesity is ineradicable.

This research studies whether an obesity epidemic can be eradicated by physical activity and how to reduce the obesity prevalence if it is ineradicable. It may have some implications on the society. First, insisting on lowering physical inactivity or its efficiency sometimes brings severe obesity prevalence. A larger physical inactive efficiency may be more effective for obesity eradication. Second, social discrimination is a coin of two sides. Making a better use of it can help people hinder obesity more effectively. In addition, based on our work, scholars can explore many other factors, like social environment, mood health, which are also very important for the epidemic. There also exist several limitations of our work. First, we lack the real-world data to verify our results. Second, social ties in the real social networks are various and have different importance; a weighted network study is a good solution.

## Methods

### The belief decision model

People’s selection for the obesity-related behaviors is absolutely voluntary and based on their mental awareness about obesity. During the spread of obesity, an individual can acquire diverse hints from his peers, including their evident appearance, regular behaviors, etc. A new piece of information can make the receiver update his mental states (i.e. tolerance for obesity), and this may result in a behavior change. Such behavior change may further change his health status and in turn produce new information for other individuals to learn. To characterize this learning process, we adopt a belief decision model which is developed based on the framework of Dempster-Shafer theory of evidence (DST). DST is widely studied in the field of Artificial Intelligence (AI) and Expert Systems, such as recognition^38^, classification^39^, fault diagnosis^40^, and also has a great potential for multi-criteria decision making^41^,^42^ which often necessitates the decision maker to make judgments on decision alternatives over a range of criteria^41^. The belief decision model in this paper is proposed to describe people’s subjective preferences for different options after they receive various information from multiple sources. We use the notion of belief to denote people’s preference for a behavior choice, and people can receive various information or hints to update their beliefs in the presence of uncertainty.

We assume that whether to take physical activity or to take physical inactivity is a dynamical binary problem, and is represented as *θ* = {*x, y*} which is called the frame of discernment in DST, where *x* represents physical activity and *y* stands for physical inactivity. People’s possible behavior responses can then be modeled as the power set of *θ*, namely 2^*θ*^ = {∅, {*x*}, {*y*}, *θ*}, where *θ* is an undecided state for “non-assigned belief” in case of inadequate knowledge or confusion^43^. People will never place any belief on the empty set. Thus there are three states for an individual, namely to be physical inactive (*x*), to be physical active (*y*) and undecided (or hesitating) for a behavior change (*θ*). The hesitating choice is used to describe the intermediate state that people are undecided for a behavior change when they receive an opposite behavior hint that deviate from their initial beliefs. If they receive more hints of the opposite behavior, their hesitation will be reduced and the preference for the deviated behavior will increase. For instance, if an individual with complete belief for physical activity receives a hint from a physical inactive peer, his physical active belief is reduced but the reduced belief is not immediately assigned to physical inactivity. Instead, he may feel a little hesitation and the belief is in fact assigned to the undecided state *θ*. After receiving a second physical inactive hint, some of his hesitating belief at state *θ* will then be transferred to physical inactivity.

Each choice is attached with a belief mass to represent people’s willingness (preference, or subjective probability) on it. We use *m*(⋅) to denote them and people’s beliefs can then be described as {*m(x*), *m(y*), *m(θ*)} which satisfies the following basic probability assignment (BPA):


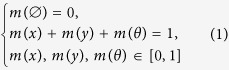


where *m(x*) denotes people’s belief mass that he should take physical inactivity, *m(y*) denotes the belief for physical activity, and *m(θ*) denotes the belief that people are hesitant to make a behavior change decision.

### Belief updating

People update their beliefs in two conditions: spontaneously updating and peer influence. First, people are unconsciously updating their beliefs based on their own health states. When a healthy person *i* becomes infected, for instance, he may form an anxious mood to return to a health state^44^ and develop a high belief for healthy behaviors. Thus we set his belief to {0, 1, 0}. But if *i* doesn’t become obese when the efficiency of physical inactivity is low, he may have a fluke mind and become more occupied in physical inactivity. We assume that he spontaneously increases physical inactive belief by receiving a new evidence of {1, 0, 0} to update his belief. For an obese person *j*, when *j* return to a healthy state by physical activity, he may develop a high belief for physical activity as {0, 1, 0} by realizing the benefits of physical activity. But a healthy weight often results from a very long time struggle with a low physical active efficiency and *j* may become disappointed. We set that if *j* doesn’t regain a healthy weight, he receives a piece of evidence of {1, 0, 0} to spontaneously increase physical inactive belief. The low efficiency of physical activity thus makes the obese people grow physical inactive belief at a high speed. Therefore, health state change and the spontaneously generated evidence while no health state change, are the sources of new information. The latter information source is very important, but is often overlooked.

Second, people’s beliefs are largely influenced by peers. As is known, people’s behaviors are very susceptible to social network effects and environmental influence^45^, such as peer influence^46^,^47^,^48^. When an obese person takes the physical inactive belief in a dominant position, his social peers are very likely to increase the similar belief and gradually become obese^49^,^50^,^51^. The spread of the physical active belief, conversely, makes people less likely to be physical inactive and reduces the obese density in the population^52^. Accordingly, the belief decision model is extended with a function for an individual to catch every change of his peers. We adopt the extended Dempster rule of combination^53^ to describe people’s belief updating process. When people receive a new piece of information or evidence, their beliefs are updated. For example, if an individual *i*, has the belief {*m*_*i*_(*x*), *m*_*i*_(*y*), *m*_*i*_(*θ*)}, his peer *j*, can receive a new piece of information from him as follows:


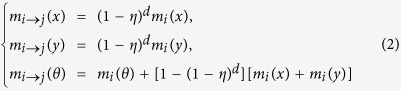


where *η* is the fading coefficient of information spread which is introduced to indicate how fast the decay is when transmitting a piece of evidence between two individuals, and *d* represents the social distance between obese and healthy people (*d* = 1 indicates no discrimination). When information spreads within the social networks, some of it is decayed because of the presence of uncertainty. The decayed belief doesn’t belong to either physical activity or inactivity, thus assigned to the “non-assigned belief” set, namely the *θ* set. After receiving the information, *j* can update his beliefs as^22^,^54^


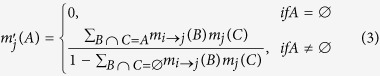


where *A, B, C* ∈ {∅, *x, y, θ*}, *x* ∩ *y* = ∅, *x* ∩ *θ* = *x* and *y* ∩ *θ* = *y*. 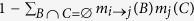 is a normalization factor.

### SIS model

**Figure 5:**
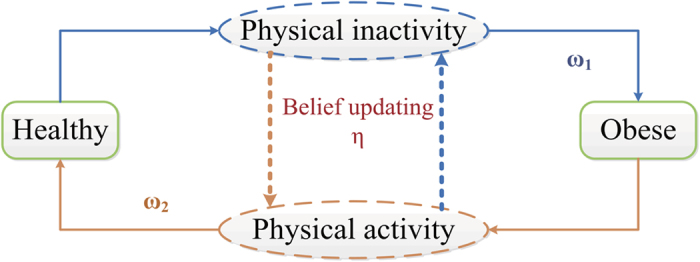
State transition diagram for the spread of obesity. Here we use “Healthy” to denote the S state, and “Obese” to denote the I state. *ω*_1_ stands for the efficiency of physical inactivity and *ω*_2_ stands for the efficiency of physical activity. *η* is the fading coefficient of information spread.

Since people normally gain or lose weight bit by bit, gaining or losing weight is usually in continuous dynamic process^1^. Moreover, there may exist several intermediate states between obese and healthy, such as overweighted, whose infectiousness is under discovering. These make it difficult to model the spread of obesity. Here we propose a simple model with the following two assumptions. First, we assume that people’s weights can be divided into discrete states. As we know, there are standards for each weight status and one weight status doesn’t overlap another. For instance, adult obesity is defined as a Body Mass Index exceeding 30. Thus it is reasonable to divide weight into several discrete states, just as the modeling of epidemic spreading^55^,^56^ and modeling of transition between different mental health states^57^. Second, we only consider two weight states: obese and nonobese (healthy), inspired by the study of Christakis^10^. On one hand, people tend to care more about the prevalence of the obesity than any other weight status. On the other hand, only obesity is proved to be infectious in present studies. Therefore, we propose a compartment model to describe the spread of obesity, as shown in Fig. 5. Healthy people will become obese by physical inactivity over a period. We set this period averaging as 1/*ω*_1_. In other words, the average efficiency of physical inactivity (or “infection rate”) is set as *ω*_1_. Obese people can only return to a health state by physical activity with an average efficiency (or “recovery rate”) of *ω*_2_. We name them physical active efficiency and physical inactive efficiency, respectively.

## Additional Information

**How to cite this article**: Huang, H. *et al*. A social contagious model of the obesity epidemic. *Sci. Rep.* **6**, 37961; doi: 10.1038/srep37961 (2016).

**Publisher's note:** Springer Nature remains neutral with regard to jurisdictional claims in published maps and institutional affiliations.
